# Comparative Efficacy Between Trifocal and Bifocal Intraocular Lens Among Patients Undergoing Cataract Surgery: A Systematic Review and Meta-Analysis

**DOI:** 10.3389/fmed.2021.647268

**Published:** 2021-09-30

**Authors:** Ziran Zhang, Haiyang Jiang, Hongwei Zhou, Fang Zhou

**Affiliations:** ^1^Department of Clinical Medicine, First Clinical Medical College, Nanjing Medical University, Nanjing, China; ^2^Department of Geriatrics, Huai'an Medical Area, Affiliated General Hospital of Eastern Theater Command, Huai'an, China; ^3^Department of Ophthalmology, The Affiliated Lianshui County People's Hospital of Kangda College of Nanjing Medical Universty, Huai'an, China; ^4^Beijing Key Laboratory of Megaregions Sustainable Development Modeling, Capital University of Economics and Business, Beijing, China; ^5^Department of Public Affairs, College of Urban Economics and Public Administration, Capital University of Economics and Business, Beijing, China

**Keywords:** cataract, trifocal intraocular lens, bifocal intraocular lens, visual acuity, meta-analysis

## Abstract

The comparative efficacy of trifocal and bifocal intraocular lenses (IOLs) remained uncertain among patients undergoing cataract surgery. A systematic review and meta-analysis was performed to answer this question. PubMed, Cochrane Library and Embase were searched to capture relevant randomized controlled trials (RCTs). Visual acuity (VA) and patient's satisfaction were regarded as primary outcomes. Secondary outcomes included residual sphere, spherical equivalence, residual cylinder, posterior capsular opacification (PCO), spectacle independence, and other complications. Statistical analysis was done using RevMan 5.2.0. A total of 9 studies (11 RCTs) with 297 participants (558 eyes) were included. Meta-analysis showed significant differences between trifocal and bifocal IOLs in the uncorrected near VA (mean difference [MD], −0.008; 95% confidence interval [Cl], −0.015 to −0.001; *P* = 0.028) and uncorrected intermediate VA (MD, −0.06; 95% CI, −0.10 to −0.02; *P* < 0.01). Trifocal IOLs were associated with decreased PCO incidence when compared to bifocal IOLs (relative risk [RR], 0.54; 95% CI, 0.31 to 0.95; *P* = 0.03). Trifocal IOLs may be superior to bifocal IOLs because of its improved intermediate VA and reduced incidence of PCO.

## Introduction

Cataract is one of the most common eye diseases in the elderly, and is also the most common reason for weakened visual performance and quality of life (1). Cataract surgery with intraocular lens (IOL) implantation has became a preferred option to restore visual acuity (VA) in these patients (2, 3). Loss of accommodative ability limited the use of monofocal IOLs, which is designed to just focus on one distance vision including VA distance vision or near vision regarding the patient's needs (4). Considering this limitation, bifocal IOLs which have the ability of dropping shadow multiple images on the retina were developed to improve the uncorrected near visual acuity (NVA) and to reduce spectacle dependence at near distance (5–7). Bifocal IOLs create two focal points for near and far distance, and thus intermediate VA is less than the near or far VA (8–10). However, intermediate vision is increasingly important because screen work has become present in nearly everybody's everyday life (4, 11). More recently, trifocal IOLs have been developed to supply the visual function at the intermediate distance (12), which is regarded as an important factor in patient satisfactory outcomes, specifically for those with extended computer use and higher patient expectations (13). Trifocal IOLs were developed in order to achieve a useful third focus for intermediate distance vision (14–16). Several comparative studies have compared the clinical outcomes of different types of multifocal IOLs, which were defined to have more than two focal points at different distances, to identify one optimal treatment option for each specific case (13, 14, 17–22). However, it remains unclear whether trifocal IOLs are superior to bifocal IOLs implantation among patients receiving cataract surgery.

To date, several published meta-analyses (1, 4, 23–25) have investigated the comparative efficacy of trifocal and bifocal IOLs. However, a conclusive finding was not generated due to several limitations such as inclusion of studies with different designs and language restriction. A recent retrospective study also revealed no significant difference between trifocal and bifocal IOLs in the uncorrected distance, intermediate, and near VA (21). Although Cruz and colleagues have published a Cochrane protocol of trifocal IOLs vs. bifocal IOLs after cataract extraction (26), and the full-text review has also been reported on June 18, 2020; (27) the reference of this review has been discounted due to the following limitations: (a) eligible patients were limited to ≥30 years with presbyopia; (b) insufficient eligible studies were included (23), and (c) pilot study which has duplicate data with subsequent formal study was considered (28). Therefore, it is necessary to update systematic reviews and meta-analysis by comprehensively investigating the comparative efficacy of trifocal and bifocal IOLs among patients receiving cataract surgery.

## Methods

This systematic review and meta-analysis was developed and performed in accordance with the methods proposed by Cochrane Collaboration (29). All results were reported based on the framework recommended by the preferred reporting items for systematic reviews and meta-analyses (PRISMA) statement (30). The structural framework of this study was developed but a formal protocol was not published.

### Literature Search

Two investigators independently searched all potentially relevant studies in PubMed, Cochrane library, and Embase from their inception until April 30, 2020. The search strategy was constructed with the combination of medical subject headings (MeSH) and text words in accordance with the requirements of individual database. The details of search strategy were documented in Supplementary File 1. Any disagreements with regarding to the literature search were resolved by a consensus principle.

### Eligibility Criteria

We mainly designed our selection criteria according to the previous meta-analysis (23). The inclusion criteria were as follows: (a) adult cataract patients aged more than 18 years who were undergoing trifocal or bifocal IOLs implantation; (b) randomized controlled trials (RCTs) investigating the comparative efficacy between trifocal and bifocal IOLs; and (c) studies that discuss at least one of the following outcomes including visual acuity (VA) (near, intermediate and distance), patient's satisfaction, residual sphere, spherical equivalence, residual cylinder, posterior capsular opacification (PCO), spectacle independence, contrast sensitivity, and complications. Studies were excluded if they met the following criteria: (a) prospective comparative study with cohort design, (b) a preliminary study group and another updated study with comprehensive information has been reported by the same study, (c) studies without sufficient information, and (d) reviews, editorials, letters, case reports, conference abstracts, and cell and animal studies. No language restriction was imposed. No ethical consent was required because this study was performed based on published data.

### Data Extraction

Two investigators independently extracted the following items using the pre-designed data extraction sheet: basic characteristics of the study including first author, publication year, and country, patients' characteristics including sample size, number of eyes, and age, and clinical characteristics of study including IOL types, outcomes, and sources of risk of bias. Visual acuity (VA) (near, intermediate and distance) and patient's satisfaction were included as primary outcomes, and the residual sphere, spherical equivalence, residual cylinder, PCO, spectacle independence, contrast sensitivity, and complications were regarded as secondary outcomes. The data that were assessed at a distance closest to 66 and 40 cm to express the near or intermediate VA were extracted. If standard deviation was estimated to be zero, then the zero value was replaced with the largest number before rounding (e.g., 0.00 to 0.0049) (4). If an included study was designed to have more than two groups, then the methods recommended by the Cochrane Handbook for Systematic Reviews of Interventions were used to divide the individual study into two unique RCTs or combine groups to create a single pair-wise comparison (29). If essential information was missing from the original study, then the leading author was contacted for additional information. Any inconsistencies in data extraction were solved based on the consensus principle.

### Quality Assessment

Two independent investigators independently assessed the quality of all eligible studies using the Cochrane risk of bias assessment tool (31) from the following items: random sequencing, allocation concealment, blinding, incomplete data, selective reporting, and other sources. An individual study was labeled as low risk if all items were fulfilled, labeled as high risk if at least one of the items was not fulfilled, and otherwise, as unclear risk. Any divergences on quality assessment were solved by consulting a third investigator.

### Statistical Analysis

Mean difference (MD) with 95% confidence interval (CI) was used to express continuous outcomes and relative risk (RR) with 95% CI was used to estimate dichotomous outcomes. For VA, residual sphere, spherical equivalence, and the residual cylinder, an MD of <0 indicates that trifocal IOL is superior over bifocal IOL. For patient's satisfaction and spectacle independence, an RR of more than 1 indicates that trifocal IOL is superior over bifocal IOL, however a RR of <1 indicates that trifocal IOL is better than bifocal IOL for PCO and other complications. The heterogeneity across studies was qualitatively assessed using Cochrane *Q* test (32), and then quantitatively estimated the level of heterogeneity with *I*^2^ statistic (33). All included studies were considered heterogeneous if *P* < 0.1 and *I*^2^> 50.0%, and otherwise regarded as homogeneous if *P* > 0.1 and *I*^2^ < 50.0% (29). All statistical analyses were performed using the random-effects model to simultaneously address variations across studies and within study (29). Moreover, subgroup analysis was also performed accordance to the IOL types. Publication bias was checked when the accumulated number of included studies for individual outcomes was more than 10 through drew funnel plot (34). Review Manager (RevMan) 5.2.0 (The Nordic Cochrane Centre, the Cochrane Collaboration, Copenhagen, Denmark) was used to complete statistical analysis. *P* < 0.05 was considered to be statistically significant difference.

## Results

### Search Results

**Figure 1:**
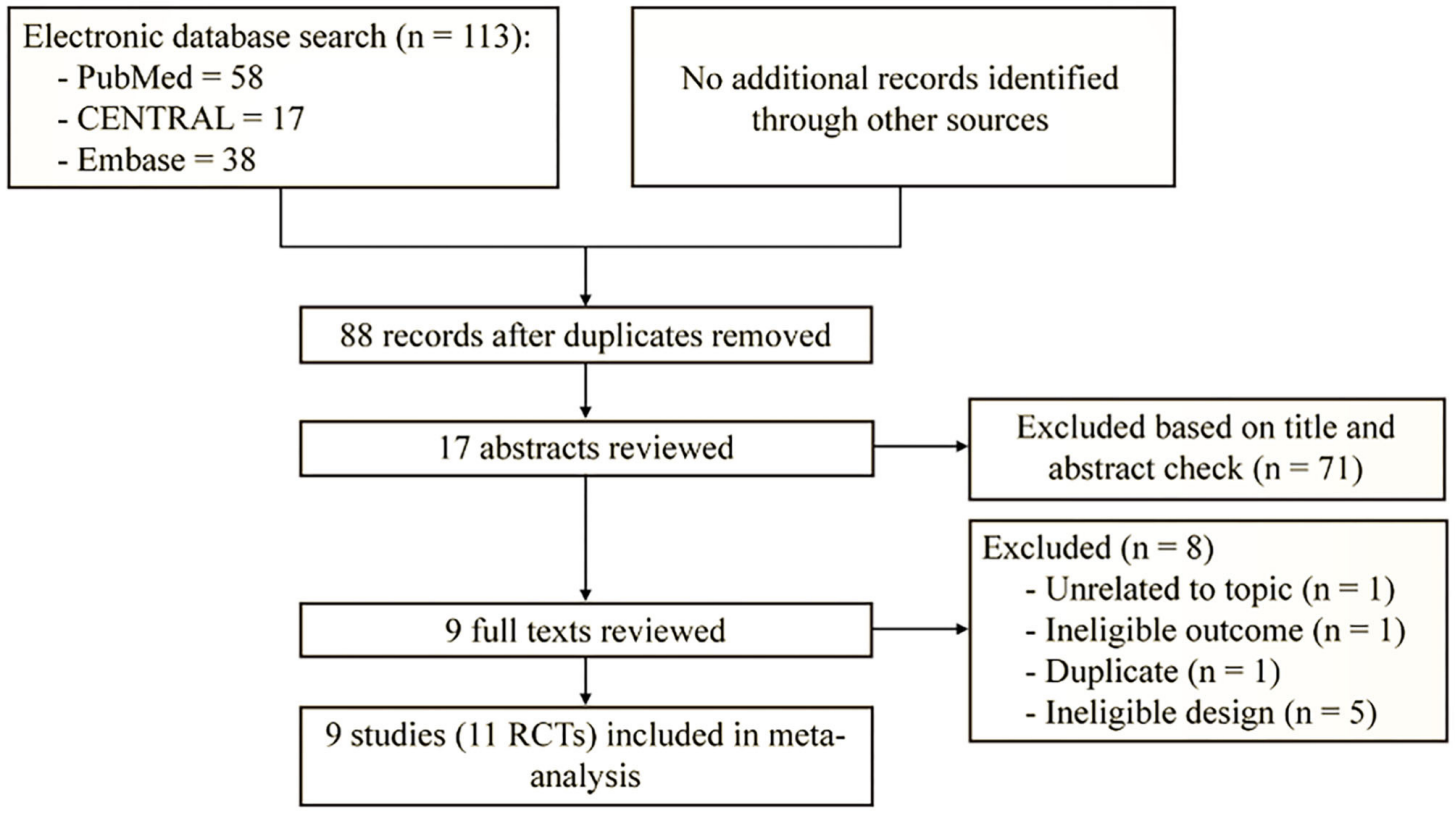
Flow diagram of retrieval and selection of literature. Other sources present reference lists of included studies.

The search and selection of potentially relevant studies were presented in Figure 1. A total of 113 records were identified after searching PubMed, Cochrane library, and Embase from their inception till April 30, 2020. Of these, 25 duplicate studies were excluded after running Find Duplication function embedded in EndNote. The title and abstract of the remaining 88 records was reviewed, and 17 studies were chosen for full-text evaluation. Finally, nine studies (8–10, 14, 17, 18, 35–37) (11 RCTs) fulfilled the inclusion criteria after excluding eight studies the following reasons: unrelated to the topic (*n* = 1), ineligible outcomes (*n* = 1), duplicate reports (*n* = 1), and ineligible study design (*n* = 5).

### Characteristics of Eligible Studies

Of the nine selected studies (11 RCTs) with 134 patients (252 eyes) in trifocal group and 163 patients (306 eyes) in bifocal group, three studies (8, 14, 17) were performed in Spain and one each in Germany (18), France (9), Norway (10), the Netherlands (35), Korea (37), and Romania (36), respectively. All studies were published between 2015 and 2018. Two studies (17, 18) were three-arm design. Seven studies (10, 14, 17, 18, 35–37) used AT LISA tri 839MP as trifocal IOLs and two (8, 9) used Fine Vision as trifocal IOLs. Three types of bifocal IOLs were used in control groups including AT LISA 809M, ReSTOR SN6AD1/2, and Tecnis ZMB00. The follow-up time ranged from 3 to 12 months. The characteristics of nine studies (11 RCTs) were outlined in Table 1.

**Table 1:** Basic characteristics of all eligible studies (11 RCTs).

**References**	**Country**	**Sample Size**		**Numbers of eyes**		**Age**		**IOL types**		**Follow-up**
		**SG**	**CG**	**SG**	**CG**	**SG**	**CG**	**SG**	**CG**	
Alió et al. (17)	Spain	8	15	16	30	63.2 ± 7.7		Trifocal IOL AT LISA tri 839MP	Bifocal IOL AT LISA 809M	12 months
Alió et al. (17)	Spain	9	17	18	34	63.2 ± 7.7		Trifocal IOL AT LISA tri 839MP	Bifocal IOL ReSTOR SN6AD1	12 months
Bilbao-Calabuig et al. (8)	Spain	12	11	24	22	56.3 ± 6.9		Trifocal IOL FineVision	Bifocal IOL ReSTOR SN6AD1/2	3 months
Cochener (9)	France	15	12	30	24	(60.6 ± 9.1)	(58.7 ± 6.4)	Trifocal IOL FineVision	Bifocal IOL Tecnis ZMB00	(5.07 ± 1.4) vs. (3.42 ± 1.16) months
Gundersen and Potvin (10, 20)	Norway	11	11	22	22	(62.1 ± 7.5)	(70.2 ± 7.8)	Trifocal IOL AT LISA tri 839MP	Bifocal IOL AT LISA 809M	3 months
Jonker et al. (35)	Netherlands	15	13	30	26	(62.6 ± 8.7)	(64.0 ± 8.8)	Trifocal IOL AT LISA tri 839MP	Bifocal IOL ReSTOR SN6AD1	3 months
Kaymak et al. (18)	Germany	7	17	14	34	(62.5 ± 6.9)	(64.4 ± 7.5)	Trifocal IOL AT LISA tri 839MP	Bifocal IOL AT LISA 809M	12 months
Kaymak et al. (18)	Germany	8	17	16	34	(62.5 ± 6.9)	(62.4 ± 8.9)	Trifocal IOL AT LISA tri 839MP	Bifocal IOL ReSTOR SN6AD1	12 months
Mojzis et al. (14)	Spain	20	18	40	35	44~70		Trifocal IOL AT LISA tri 839MP	Bifocal IOL AT LISA 809M	12 months
Mojzis et al. (37)	Korea	20	23	24	27	(49.5 ± 6.7)	(52.4 ± 9.3)	Trifocal IOL AT LISA tri 839MP	Bifocal IOL ReSTOR SN6AD1	3 months
Postolache and Postolache (36)	Bacau	9	9	18	18	n.r.		Trifocal IOL AT LISA tri 839MP	Bifocal IOL AT LISA 809M	6 months

*IOL, intraocular lens; SG, study group representing trifocal IOLs; CG, control group indicating bifocal IOLs; n.r., not reported*.

### Risk of Bias

**Figure 2:**
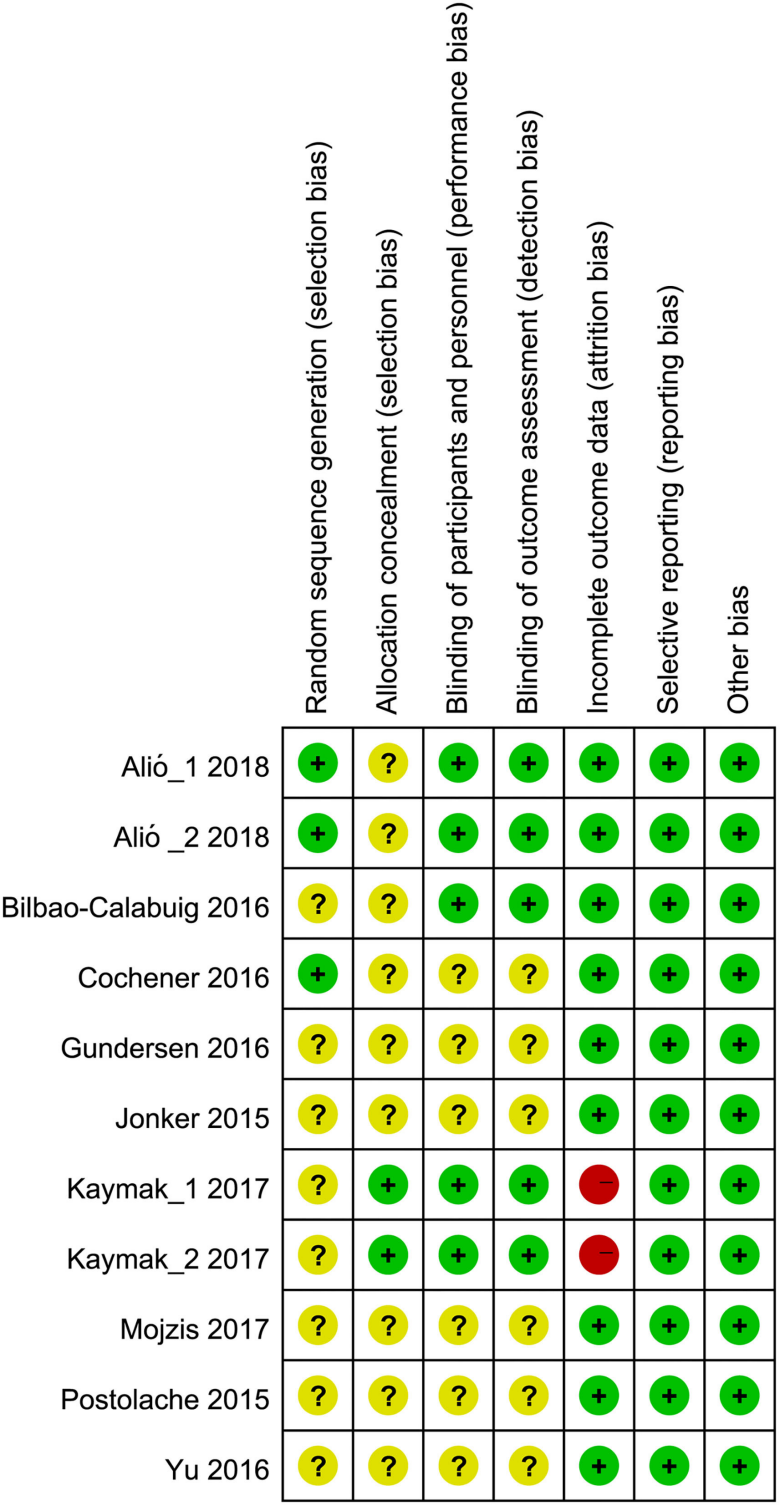
Risk of bias summary. Green, yellow and red solid circles represented low, unclear and high risk of bias.

Among the nine studies, only two studies (9, 17) reported the details of random sequencing, one study (18) appropriately performed allocation concealment, three studies (8, 17, 18) blinded the participants and outcomes assessors, one study (18) has missing data and did not report the reasons for drop-out, and all studies have low risk in the remaining items. Generally, one study (2 RCTs) (18) was graded as high risk of bias, and the remaining eight studies (9 RCTs) (8–10, 14, 17, 35–37) had unclear risk of bias (Figure 2).

### Primary Outcomes

#### Visual Acuity: Near Visual Acuity

**Figure 3:**
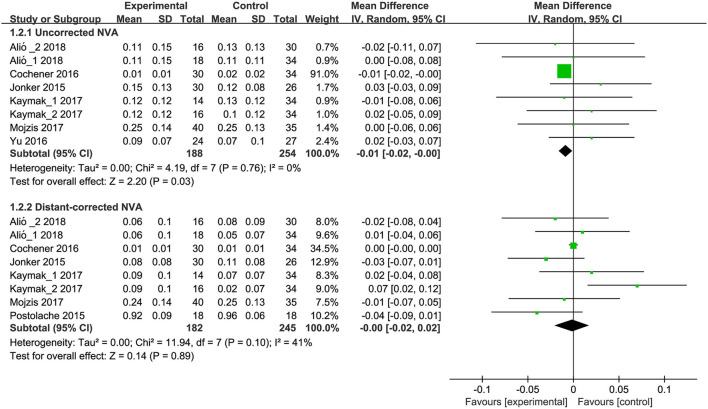
Meta-analysis of NVA between trifocal and bifocal IOLs. SD, standard difference; IV, inverse variance; CI, confidence interval; NVA, near visual acuity. Experimental and control indicate trifocal and bifocal IOLs, respectively.

Among the nine included studies, six studies (9, 14, 17, 18, 35, 36) including 8 RCTs reported uncorrected near visual acuity (NVA), and meta-analysis indicated a significant difference between trifocal and bifocal IOLs implantation (MD, −0.008; 95% CI, −0.015 to −0.001, *P* = 0.028; Figure 3). Moreover, six studies (9, 14, 17, 18, 35, 36) (8 RCTs) reported distant-corrected NVA, and pooled results suggested no significant differences between trifocal and bifocal IOLs implantation (MD, −0.00; 95% CI, −0.02 to 0.02, *P* = 0.89; Figure 3). Subgroup analyses results revealed that trifocal IOLs with FineVision was greater than bifocal IOLs (1 RCT, MD, −0.01; 95% CI, −0.018 to −0.002, *P* = 0.010; Supplementary Figure S1) for uncorrected NVA. However, other comparisons showed no significant differences in uncorrected (Supplementary Figure S1) and distant-corrected NVA (Supplementary Figure S2).

#### Visual Acuity: Intermediate Visual Acuity

**Figure 4:**
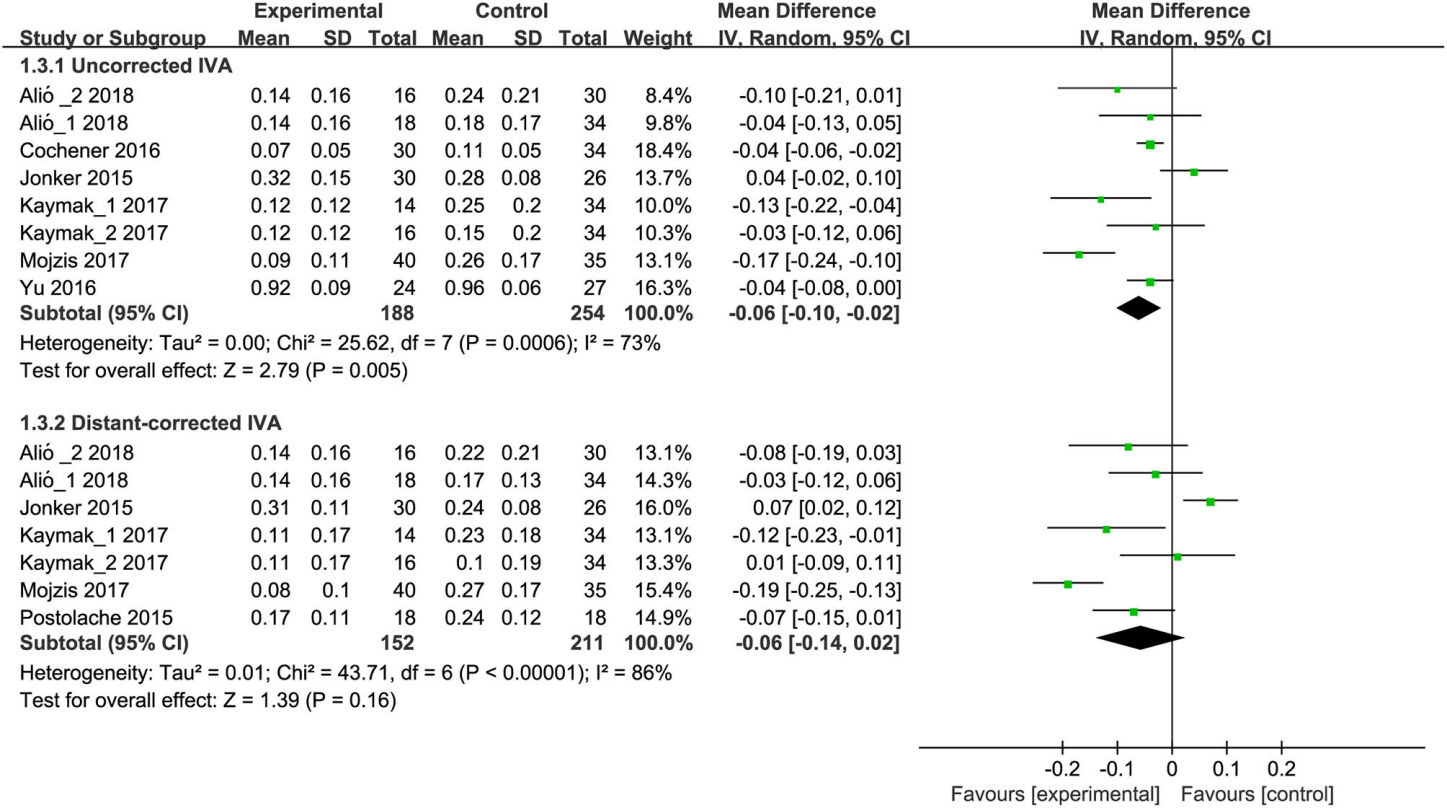
Meta-analysis of IVA between trifocal and bifocal IOLs. SD, standard difference; IV, inverse variance; CI, confidence interval; IVA, intermediate visual acuity. Experimental and control indicate trifocal and bifocal IOLs, respectively.

Six eligible studies (9, 14, 17, 18, 35, 37) (8 RCTs) have reported uncorrected intermediate VA (IVA). Meta-analysis results revealed that trifocal IOLs were linked with improved uncorrected IVA when compared to bifocal IOLs (MD, −0.06; 95% CI, −0.10 to −0.02; *P* < 0.01; Figure 4). Five studies (14, 17, 18, 35, 36) including 7 RCTs reported distant-corrected IVA, and meta-analysis results showed a significant difference between trifocal and bifocal IOLs (MD, −0.06; 95% CI, −0.14 to 0.02; *P* = 0.16; Figure 4). Subgroup analysis showed a significant difference between trifocal IOL with AT LISA tri 839MP and bifocal IOL with AT LISA 809M for uncorrected (MD, −0.12; 95% CI, −0.19 to −0.04; *P* < 0.01; Supplementary Figure S3) and distant-corrected IVA (MD, −0.10; 95% CI, −0.18 to −0.03; *P* < 0.01; Supplementary Figure S4). Moreover, subgroup analysis also revealed that trifocal IOLs with FineVision showed association with improved uncorrected IVA when compared to bifocal IOLs (1 RCT; MD, −0.04; 95% CI, −0.06 to −0.02; *P* < 0.01; Supplementary Figure S3). Other comparisons were undetected and showed a significant difference in uncorrected (Supplementary Figure S3) and distant-corrected IVA (Supplementary Figure S4).

#### Visual Acuity: Distant Visual Acuity

**Figure 5:**
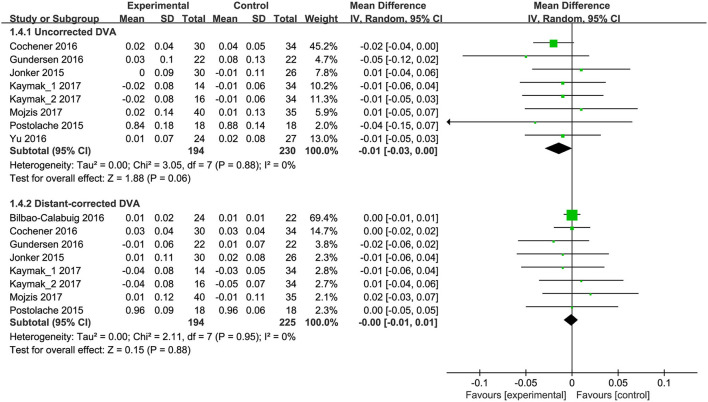
Meta-analysis of DVA between trifocal and bifocal IOLs. SD, standard difference; IV, inverse variance; CI, confidence interval; DVA, distant visual acuity. Experimental and control indicate trifocal and bifocal IOLs, respectively.

Of the 9 eligible studies, seven studies (9, 10, 14, 18, 35–37) (8 RCTs) reported uncorrected distant VA (DVA). Meta-analysis results showed no significant difference between trifocal and bifocal IOLs for uncorrected DVA (MD, −0.014; 95% CI, −0.029 to 0.001; *P* = 0.06; Figure 5). Seven studies (8–10, 14, 18, 35, 36) (8 RCTs) reported distant-corrected DVA, and meta-analysis also showed no significant difference between trifocal and bifocal IOLs (MD, −0.00; 95% CI, −0.01 to 0.01; *P* = 0.88; Figure 5). Moreover, all subgroup analyses results based on IOL types showed no statistically significant differences for uncorrected (Supplementary Figure S5) and distant-corrected DVA (Supplementary Figure S6) between trifocal and bifocal IOLs.

#### Patient's Satisfaction

**Figure 6:**
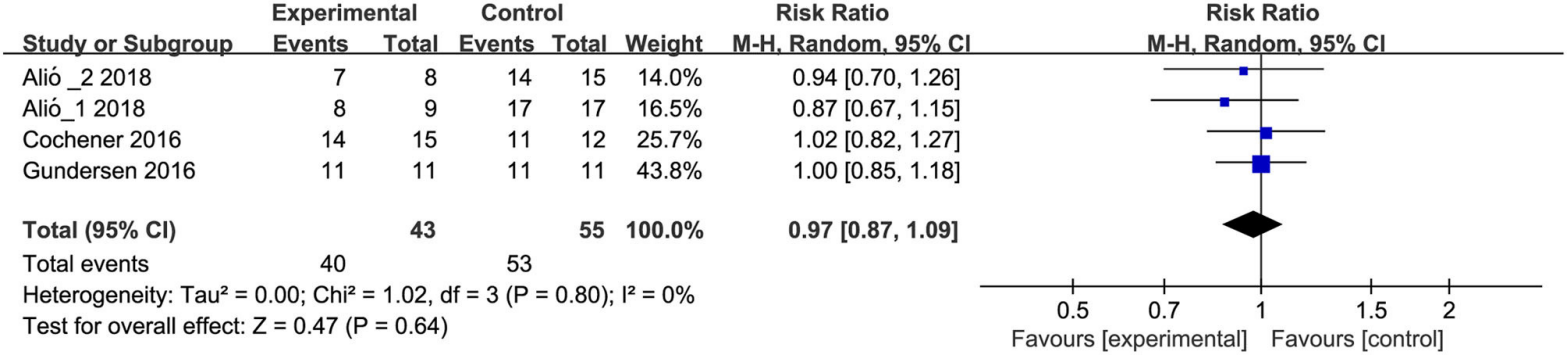
Meta-analysis of patient's satisfaction between trifocal and bifocal IOLs. M-H, Mantel-Haenszel; CI, confidence interval. Experimental and control indicate trifocal and bifocal IOLs, respectively.

Three studies (9, 10, 17) with 43 eyes in trifocal IOLs group and 55 eyes in bifocal IOLs group reported patient's satisfaction. Meta-analysis results suggested no significant differences between trifocal and bifocal IOLs with regard to patient's satisfaction (RR, 0.97; 95% CI, 0.87 to 1.09; *P* = 0.64; Figure 6). Moreover, one study (35) considered patient's satisfaction as an outcome, but no numerical data were obtained from the original study. This study also showed no significant difference between trifocal and bifocal IOLs with regard to patient's satisfaction.

### Secondary Outcomes

(a) Qualitative variables: among the nine included studies, two studies (9, 14), five studies (8, 10, 14, 35, 37), four studies (9, 10, 14, 35), two studies (3 RCTs) (14, 17), two studies (9, 35), and three studies (9, 10, 35) reported residual sphere, spherical equivalence, residual cylinder, PCO, spectacle independence, and other complications. Meta-analysis results revealed that trifocal IOLs have significantly decreased the PCO incidence when compared to bifocal IOLs (RR, 0.54; 95% CI, 0.31 to 0.95; *P* = 0.03; Supplementary Figure S7). However, there was no significant difference between trifocal and bifocal IOLs in the residual sphere, spherical equivalence, residual cylinder, spectacle independence, and other complications (Supplementary Figure S7). (b) Quantitative variables: among nine eligible studies, seven studies (8–10, 14, 17, 35, 37) reported contrast sensitivity as outcome. However, no numerical data served to perform meta-analysis, and thus these results were descriptively summarized. Five studies (8–10, 14, 17) suggested that trifocal IOLs showed no improvement in the contrast sensitivity when compared to bifocal IOLs, however the remaining two studies (35, 37) found that bifocal IOLs were better than trifocal IOLs in improving contrast sensitivity. With these conflicting results, the comparative efficacy of trifocal and bifocal IOLs with regard to contrast sensitivity cannot be conclusively determined.

## Discussion

### Main Findings

Multifocal IOLs were extensively used to deliver functional uncorrected vision for over a range of distances (10, 35). The most commonly used multifocal IOLs in clinical practice are bifocal and trifocal IOLs (23). Although several clinical trials (17, 18, 35) and meta-analyses (1, 4, 23–25, 27) have been conducted to investigate the comparative efficacy between trifocal and bifocal IOLs, a definitive conclusion has not yet been achieved.

In this updated systematic review and meta-analysis, we included 9 studies including 11 RCTs with 134 patients (252 eyes) in trifocal IOL group and 163 patients (306 eyes) in bifocal IOL group finally. We obtained four main findings after performing this meta-analysis. Firstly, trifocal IOLs showed an improvement in the uncorrected near VA after cataract surgery and uncorrected intermediate VA when compared to bifocal IOLs. However the result about near VA should be cautiously interpreted because the clinical relevance of near visual acuity is defined as a MD of −0.01. Secondly, trifocal IOLs decreased the incidence of PCO when compared to bifocal IOLs because the most trifocal IOLs design an anti-PCO posterior profile to reinforce the effect of the square edges in preventing PCO formation (17). Thirdly, trifocal IOL with AT LISA tri 839MP was superior to bifocal IOL with LISA 809M in improving uncorrected and distant-corrected intermediate VA. Fourthly, there was no significant differences between trifocal and bifocal IOLs for uncorrected distant VA and distant-corrected near VA, intermediate VA, and distant VA, patient's satisfaction, residual sphere, spherical equivalence, residual cylinder, spectacle independence, and other complications. Moreover, the role of trifocal IOLs in improving contrast sensitivity still remained inconclusive when compared to bifocal IOLs. Meanwhile, the lack of statistically significant difference in terms of patient's satisfaction may attribute to the fact that trifocal and bifocal IOLs all showed excellent performance of spectacle independence, which is in line with the results of previous meta-analysis (23). Certainly, different questionnaires such as self-designed in-house questionnaire and VF-14 questionnaire used in individual study may be the contributing to this result (23).

### Comparisons of the Present Study and Previous Meta-Analyses

To date, there are six meta-analyses had been published to investigate comparative efficacy between trifocal and bifocal IOLs. In 2017, Shen and colleagues included four RCTs and four cohorts to perform a meta-analysis for the purpose of investigating patient outcomes following implantation of trifocal or bifocal IOLs (1), and the results revealed that patients receiving trifocal IOLs had better intermediate VA than those receiving bifocal IOLs. In this meta-analysis study, authors incorporated studies with different designs into an analysis unit and did not perform subgroup analysis according to study design, limiting the reliability of pooled results. Following the previous study, Xu and colleagues performed another meta-analysis to determine the clinical performance between trifocal and bifocal IOLs, and the results indicated that trifocal IOLs (especially AT Lisa trifocal 839M trifocal) demonstrated a clear advantage over bifocal IOLs in intermediate VA (24). This study included 6 RCTs and two cohort studies for statistical analysis. However, subgroup analysis based on study design was not carried out, and so the conclusions must be cautiously interpreted. In the same year, Yoon and colleagues also conducted a meta-analysis that compared the efficacy between trifocal and bifocal IOLs implantation after cataract surgery or refractive lens (4). The results of this study suggested that trifocal IOLs implantation is superior over bifocal IOLs in intermediate VA. Unfortunately, incorporation of studies with different study designs in individual synthesis compromised the reliability of findings. In 2018, Yang et al., evaluated comparative efficacy of trifocal and bifocal IOLs in patients receiving phacoemulsification using meta-analysis, and found similar levels of monocular distance and near VA between trifocal and bifocal IOLs, and this was inconsistent with that of the previous findings (25). It is noted that 4 RCTs and four cohorts were included for the final analysis, but subgroup analysis was not considered. After careful review of the four aforementioned meta-analyses, Jin and colleagues performed a meta-analysis of RCTs to compare the clinical performance of bifocal and trifocal IOLs in cataract surgery (23). This study included 8 RCTs for the final analysis and suggested that trifocal IOLs, especially AT LISA tri 839M, is superior over bifocal IOLs for intermediate VA. However, a prospective cohort and a duplicate preliminary study performed by the same group were included, and an eligible study with three-arm was missed. These drawbacks might limit the reliability and robustness of the summarized results. In the present meta-analysis, nine prospective comparative studies that were divided into 11 RCTs were included for the final analysis and found that trifocal IOLs are superior to bifocal IOLs for uncorrected near VA, uncorrected intermediate VA, and PCO incidence. Meanwhile, a subgroup analysis was also designed according to the IOL types and found that trifocal IOL with LISA tri 839MP demonstrated better uncorrected and distant-corrected intermediate VA than bifocal IOL with LISA 809M. In 2020, Zamora-de La Cruz D and colleagues reported the full-text of a previous Cochrane protocol focusing the comparative efficacy of trifocal vs. bifocal IOLs among participants with presbyopia undergoing cataract extraction. Although this review completely followed the requirements proposed by the Cochrane network, however some limitations still impair the reference of conclusion: (a) narrower eligible patients (≥30 years with presbyopia); (b) did not include all eligible studies, and (c) included preliminary study which has duplicate data with subsequent formal study was considered.

In the present study, we included more studies to generate more reliable findings. However, several limitations must be acknowledged. First, subgroup analysis was not performed to explore the impact of follow-up on pooled results due to the limited number of eligible studies. So, it is necessary to further investigate the time effect of trifocal and bifocal IOLs in the future studies. Second, funnel plot was not drawn to inspect the publication bias due to insufficient number of eligible studies. However, language restriction was not imposed to capture all potentially relevant studies. Third, most of the eligible studies did not report the details of the risk of bias, limiting our ability to appraise the levels of evidence. Therefore, future studies with rigorous methodology should be designed. Fourth, significant heterogeneity was detected for some outcomes, which might be due to small sample size of each included study, and thus large-scale study is further warranted.

In summary, the present systematic review and meta-analysis suggested that patients receiving trifocal IOLs have better uncorrected near and intermediate VA and lower incidence of PCO when compared with those receiving bifocal IOLs. But the uncorrected distant VA and distant-corrected near VA, intermediate VA, and distant VA, patient's satisfaction, residual sphere, spherical equivalence, residual cylinder, spectacle independence, and other complications of bifocal IOLs were similar to those of trifocal IOLs. Moreover, this study also revealed that trifocal AT LISA tri 839M showed association with improved intermediate VA when compared to bifocal LISA 809M.

## Data Availability Statement

The original contributions presented in the study are included in the article/Supplementary Material, further inquiries can be directed to the corresponding authors.

## Author Contributions

HZ conceived the study. HJ and ZZ captured and selected citations. HZ and ZZ designed the data extraction table. ZZ extracted data. FZ performed all statistical analyses and prepared the manuscript draft. HZ and FZ revised the initial manuscript and critically edited language. All authors approved the final version of the manuscript.

## Funding

This work was funded by 2018 Jiangsu Province PHD of Innovation and Entrepreneurship Project, 333 High-level Talent Training Project in Jiangsu Province [(2018) III-1063] and Lianshui County Science and Technology Bureau Project (2020).

## Conflict of Interest

The authors declare that the research was conducted in the absence of any commercial or financial relationships that could be construed as a potential conflict of interest.

## Publisher's Note

All claims expressed in this article are solely those of the authors and do not necessarily represent those of their affiliated organizations, or those of the publisher, the editors and the reviewers. Any product that may be evaluated in this article, or claim that may be made by its manufacturer, is not guaranteed or endorsed by the publisher.

## Supplementary Material

Supplementary File 1 Search strategies by targeted databases including PubMed, Cochrane Library, and Embase.

Supplementary Figure S1 Subgroup analysis of uncorrected NVA according to IOL types. SD, standard difference; IV, inverse variance; CI, confidence interval; NVA, near visual acuity; IOL, intraocular lens. Experimental and control indicate trifocal and bifocal IOLs, respectively.

Supplementary Figure S2 Subgroup analysis of distant-corrected NVA according to IOL types. SD, standard difference; IV, inverse variance; CI, confidence interval; NVA, near visual acuity; IOL, intraocular lens. Experimental and control indicate trifocal and bifocal IOLs, respectively.

Supplementary Figure S3 Subgroup analysis of uncorrected IVA according to IOL types. SD, standard difference; IV, inverse variance; CI, confidence interval; IVA, intermediate visual acuity; IOL, intraocular lens. Experimental and control indicate trifocal and bifocal IOLs, respectively.

Supplementary Figure S4 Subgroup analysis of distant-corrected IVA according to IOL types. SD, standard difference; IV, inverse variance; CI, confidence interval; IVA, intermediate visual acuity; IOL, intraocular lens. Experimental and control indicate trifocal and bifocal IOLs, respectively.

Supplementary Figure S5 Subgroup analysis of uncorrected DVA according to IOL types.SD, standard difference; IV, inverse variance; CI, confidence interval; DVA, distant visual acuity; IOL, intraocular lens. Experimental and control indicate trifocal and bifocal IOLs, respectively.

Supplementary Figure S6 Subgroup analysis of distant-corrected DVA according to IOL types. SD, standard difference; IV, inverse variance; CI, confidence interval; DVA, distant visual acuity; IOL, intraocular lens. Experimental and control indicate trifocal and bifocal IOLs, respectively.

Supplementary Figure S7 Meta-analysis of secondary outcomes including residual sphere **(A)**, spherical equivalence **(B)**, residual cylinder **(C)**, PCO **(D)**, spectacle independence **(E)**, and complications **(F)**. M-H, Mantel-Haenszel; IV, inverse variance; CI, confidence interval; PCO, posterior capsular opacification. Experimental and control indicate trifocal and bifocal IOLs, respectively.
