# Supplementary material for: Comparative Efficacy Between Trifocal and Bifocal Intraocular Lens Among Patients Undergoing Cataract Surgery: A Systematic Review and Meta-Analysis
Source: Front Med (Lausanne). 2021 Sep 30;8:647268. doi: 10.3389/fmed.2021.647268 (PMC8514957; doi:10.3389/fmed.2021.647268)
Supplement: Supplementary File 1 — Search strategies by targeted databases including PubMed, Cochrane Library, and Embase. [file Table_1.DOCX]

**1. PubMed search strategy**

| Search number | Search Details |
| --- | --- |
| 6 | (((("lenses, intraocular"[MeSH Terms] OR "lens implantation, intraocular"[MeSH Terms]) OR "Phakic Intraocular Lenses"[MeSH Terms]) OR (((((("intraocular lenses"[Title/Abstract] OR "intraocular lens"[Title/Abstract]) OR "implantable contact lens"[Title/Abstract]) OR "intraocular lens implantation"[Title/Abstract]) OR "intraocular lens implantations"[Title/Abstract]) OR "phakic intraocular lens"[Title/Abstract]) OR "Phakic Intraocular Lenses"[Title/Abstract])) AND "trifocal"[Title/Abstract]) AND "bifocal"[Title/Abstract] |
| 5 | "bifocal"[Title/Abstract] |
| 4 | "trifocal"[Title/Abstract] |
| 3 | "lenses, intraocular"[MeSH Terms] OR "lens implantation, intraocular"[MeSH Terms] OR "Phakic Intraocular Lenses"[MeSH Terms] OR "intraocular lenses"[Title/Abstract] OR "intraocular lens"[Title/Abstract] OR "implantable contact lens"[Title/Abstract] OR "intraocular lens implantation"[Title/Abstract] OR "intraocular lens implantations"[Title/Abstract] OR "phakic intraocular lens"[Title/Abstract] OR "Phakic Intraocular Lenses"[Title/Abstract] |
| 2 | "intraocular lenses"[Title/Abstract] OR "intraocular lens"[Title/Abstract] OR "implantable contact lens"[Title/Abstract] OR "intraocular lens implantation"[Title/Abstract] OR "intraocular lens implantations"[Title/Abstract] OR "phakic intraocular lens"[Title/Abstract] OR "phakic intraocular lenses"[Title/Abstract] |
| 1 | "lenses, intraocular"[MeSH Terms] OR "lens implantation, intraocular"[MeSH Terms] OR "Phakic Intraocular Lenses"[MeSH Terms] |

**2. Cochrane library search strategy**

**ID Search**

#1 (Intraocular Lenses):ti,ab,kw OR (Intraocular Lens):ti,ab,kw OR (Implantable Contact Lens):ti,ab,kw OR (Intraocular Lens Implantation):ti,ab,kw OR (Intraocular Lens Implantations):ti,ab,kw (Word variations have been searched)

#2 (Phakic Intraocular Lenses):ti,ab,kw OR (Phakic Intraocular Lens):ti,ab,kw (Word variations have been searched)

#3 #1 OR #2

#4 MeSH descriptor: [Lenses, Intraocular] explode all trees

#5 MeSH descriptor: [Lens Implantation, Intraocular] explode all trees

#6 MeSH descriptor: [Phakic Intraocular Lenses] explode all trees

#7 #3 OR #4 OR #5 OR #6

#8 (trifocal):ti,ab,kw (Word variations have been searched)

#9 (bifocal):ti,ab,kw (Word variations have been searched)

#10 #7 AND #8 AND #9

**3. Embase search strateg**y

**No. Query**

#12. #4 AND #7 AND #10 AND #11

#11. random*

#10. #7 OR #8 OR #9

#9. 'bifocal lens'/exp OR 'bifocal contact lens'/exp

#8. bifocal:ti,ab,kw

#7. #5 OR #6

#6. 'trifocal intraocular lens'/exp

#5. trifocal:ti,ab,kw

#4. #1 OR #2 OR #3

#3. 'phakic intraocular lens'/exp

#2. 'lens implant'/exp

#1. 'intraocular lenses':ti,ab,kw OR 'intraocular lens':ti,ab,kw OR 'implantable contact lens':ti,ab,kw OR 'intraocular lens implantation':ti,ab,kw OR 'intraocular lens implantations':ti,ab,kw OR 'phakic intraocular lenses':ti,ab,kw OR 'phakic intraocular lens':ti,ab,kw

.......................................................
