# Supplementary figures and images for: Comparative Efficacy Between Trifocal and Bifocal Intraocular Lens Among Patients Undergoing Cataract Surgery: A Systematic Review and Meta-Analysis
Source: Front Med (Lausanne). 2021 Sep 30;8:647268. doi: 10.3389/fmed.2021.647268 (PMC8514957; doi:10.3389/fmed.2021.647268)

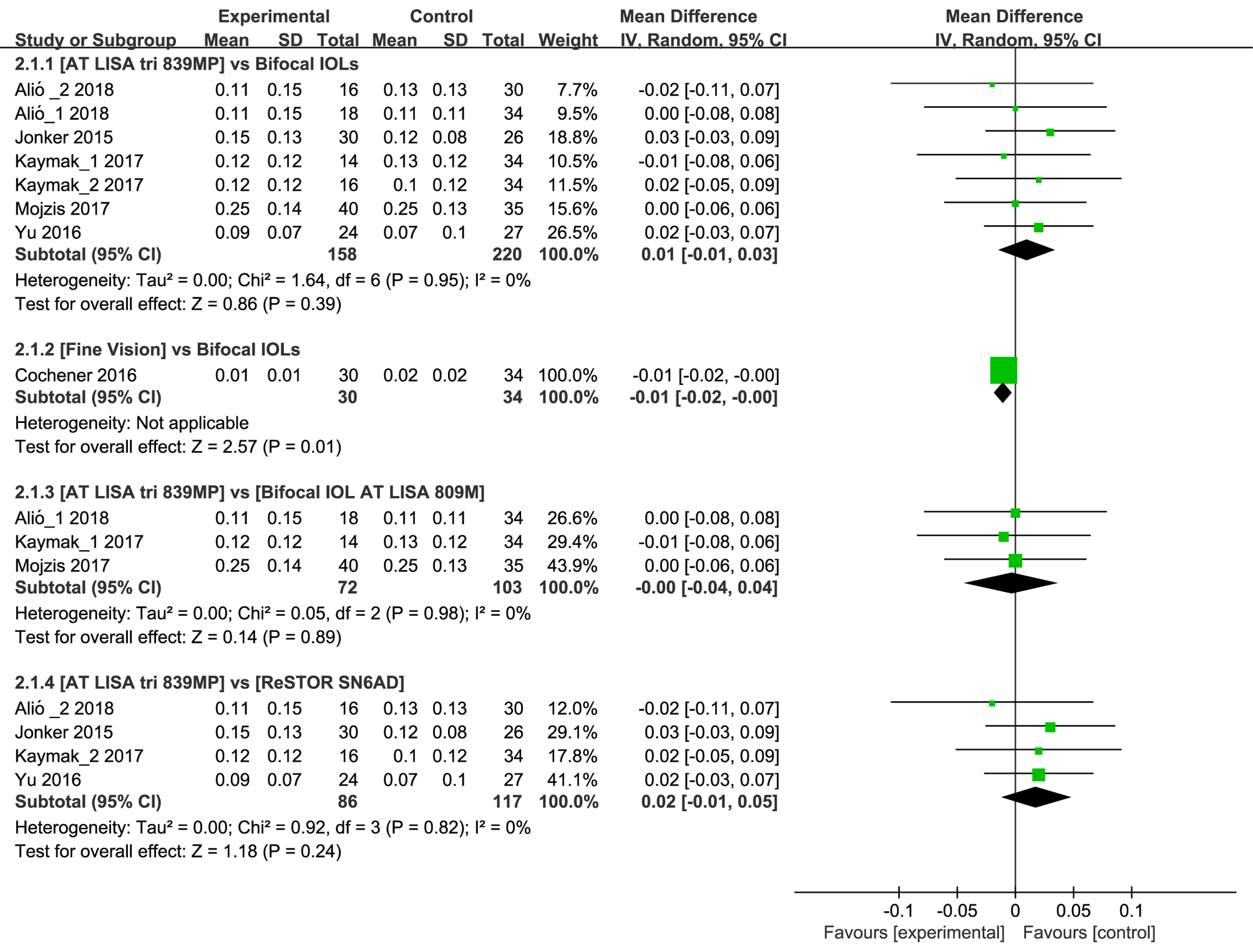

Supplement: Supplementary Figure S1 — Subgroup analysis of uncorrected NVA according to IOL types. SD, standard difference; IV, inverse variance; CI, confidence interval; NVA, near visual acuity; IOL, intraocular lens. Experimental and control indicate trifocal and bifocal IOLs, respectively. [file Image_1.TIF]

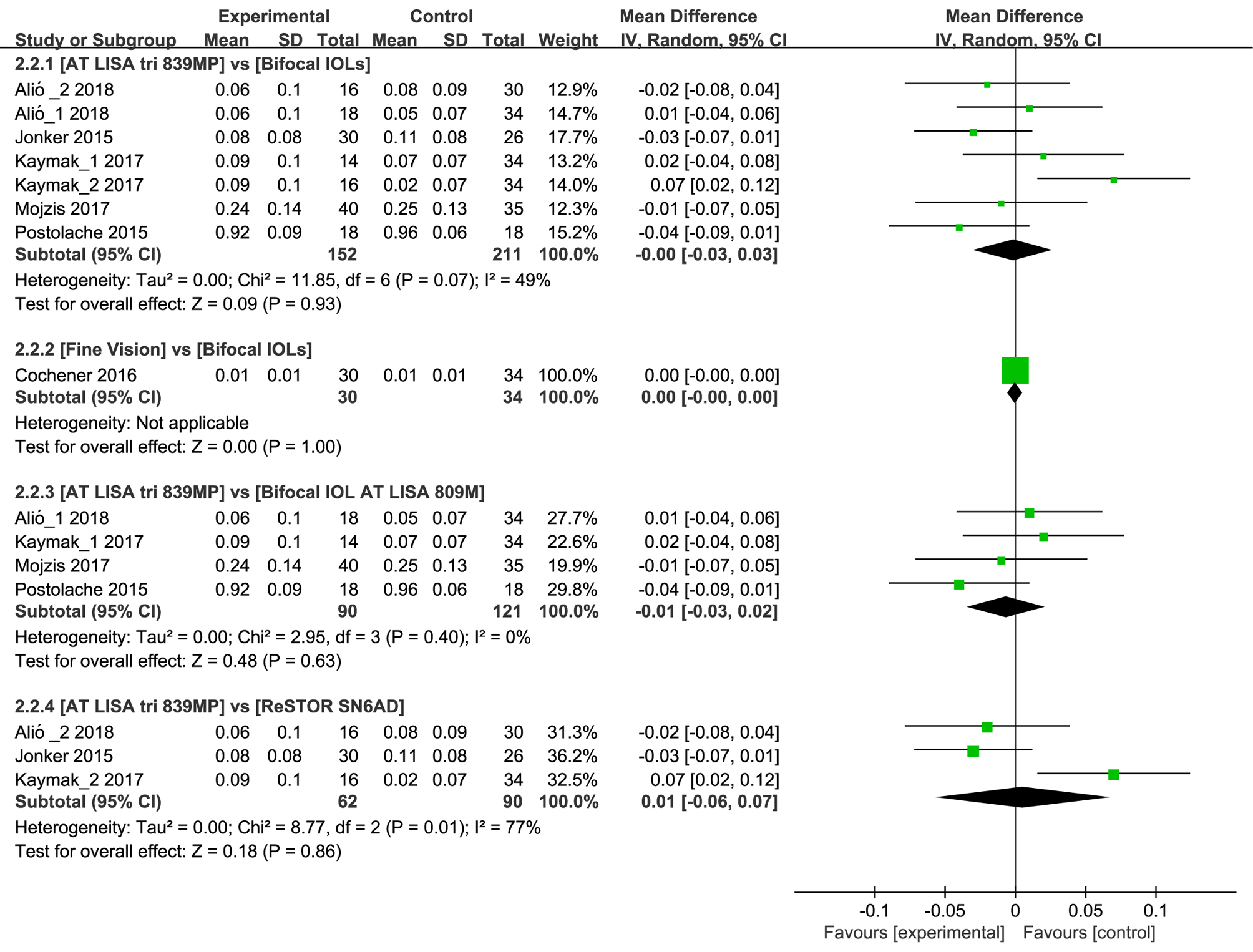

Supplement: Supplementary Figure S2 — Subgroup analysis of distant-corrected NVA according to IOL types. SD, standard difference; IV, inverse variance; CI, confidence interval; NVA, near visual acuity; IOL, intraocular lens. Experimental and control indicate trifocal and bifocal IOLs, respectively. [file Image_2.TIF]

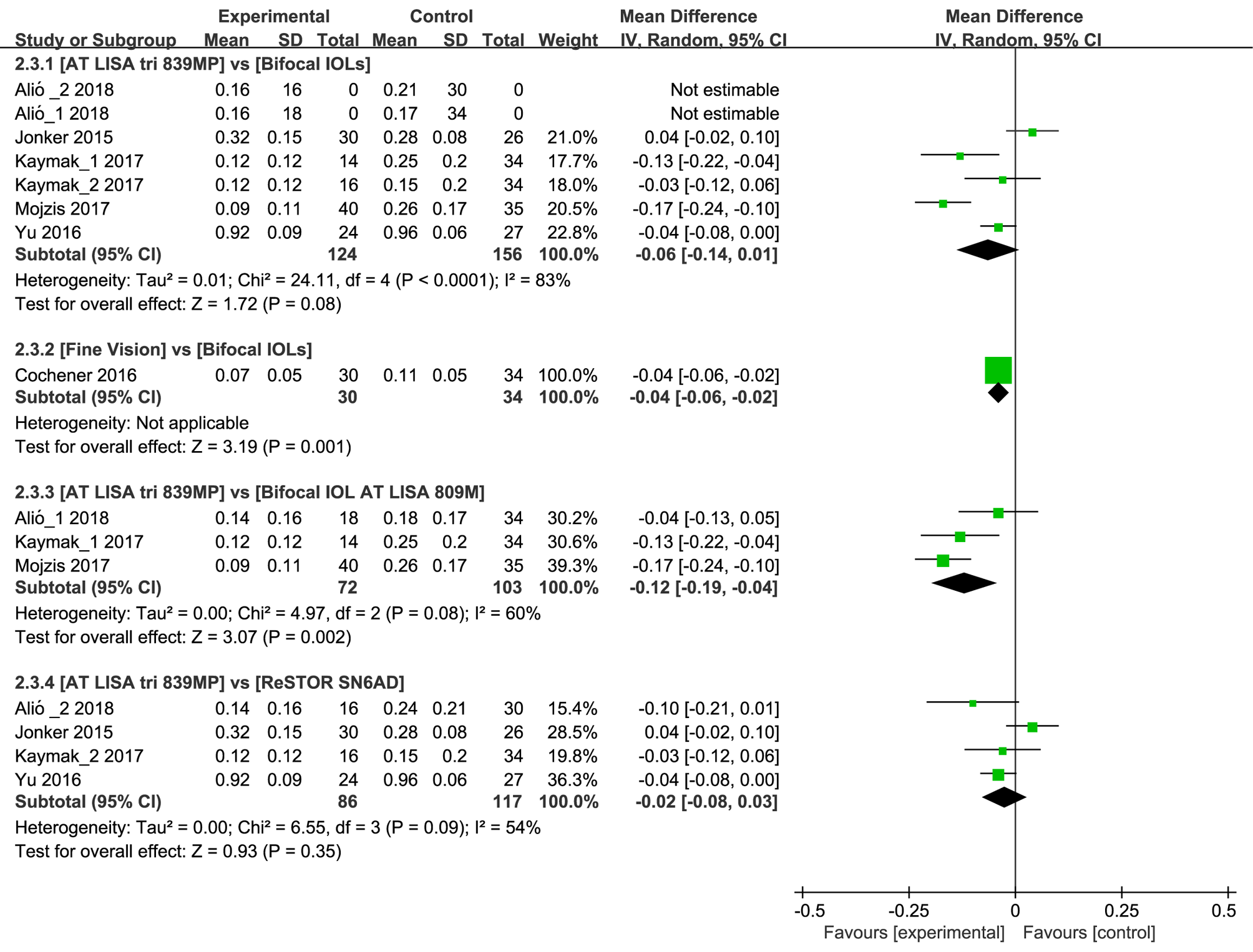

Supplement: Supplementary Figure S3 — Subgroup analysis of uncorrected IVA according to IOL types. SD, standard difference; IV, inverse variance; CI, confidence interval; IVA, intermediate visual acuity; IOL, intraocular lens. Experimental and control indicate trifocal and bifocal IOLs, respectively. [file Image_3.TIF]

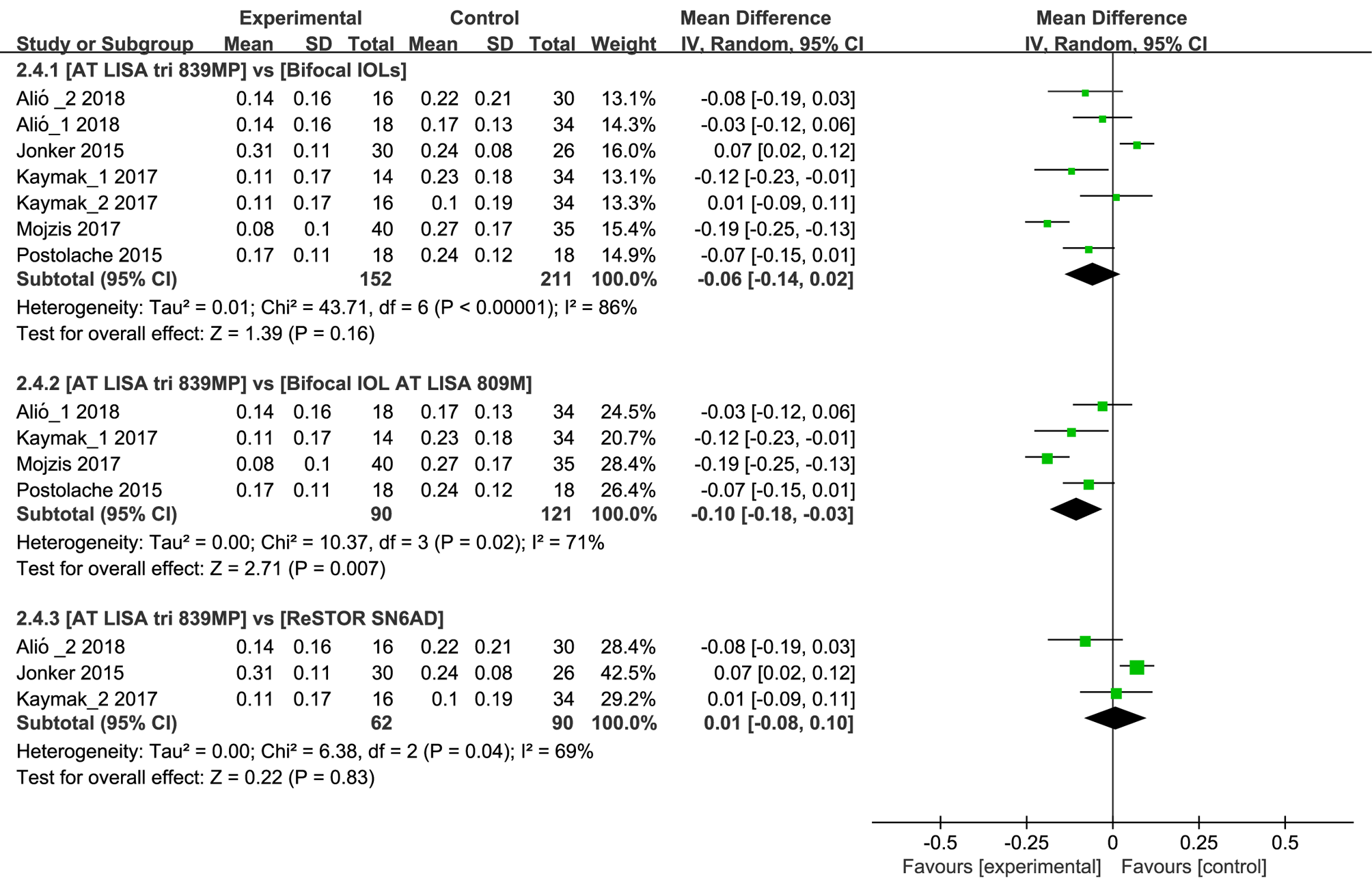

Supplement: Supplementary Figure S4 — Subgroup analysis of distant-corrected IVA according to IOL types. SD, standard difference; IV, inverse variance; CI, confidence interval; IVA, intermediate visual acuity; IOL, intraocular lens. Experimental and control indicate trifocal and bifocal IOLs, respectively. [file Image_4.TIF]

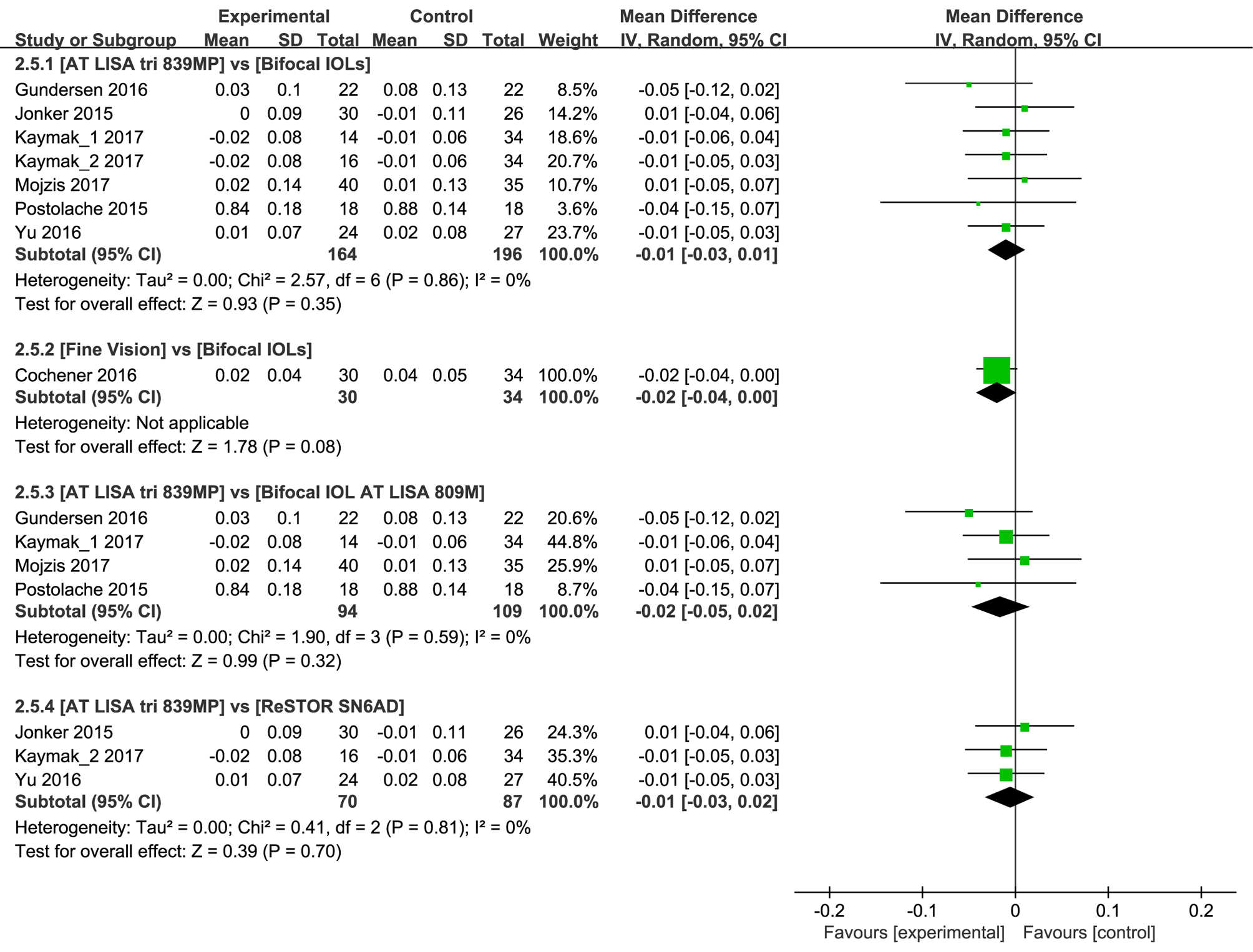

Supplement: Supplementary Figure S5 — Subgroup analysis of uncorrected DVA according to IOL types.SD, standard difference; IV, inverse variance; CI, confidence interval; DVA, distant visual acuity; IOL, intraocular lens. Experimental and control indicate trifocal and bifocal IOLs, respectively. [file Image_5.TIF]

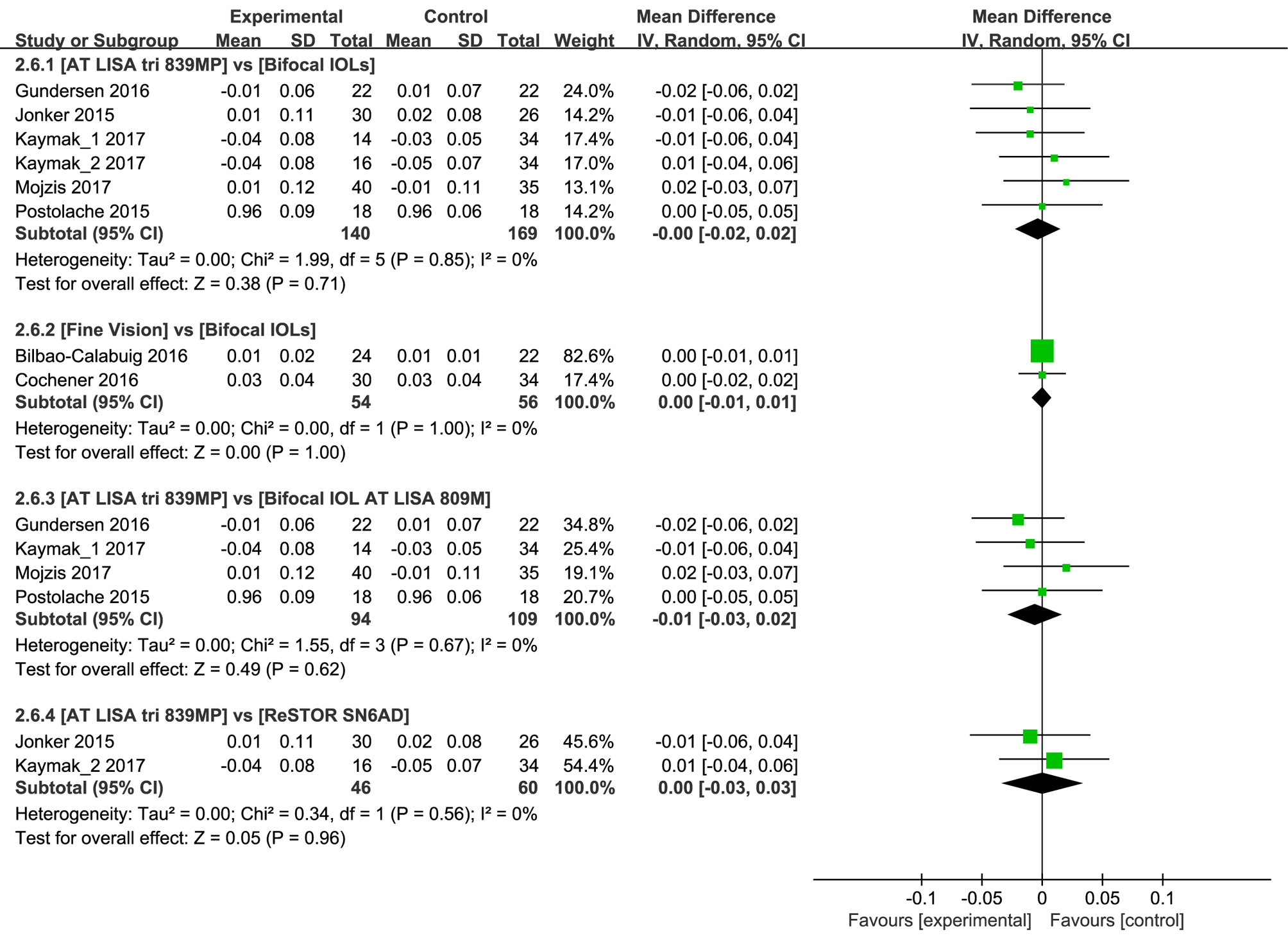

Supplement: Supplementary Figure S6 — Subgroup analysis of distant-corrected DVA according to IOL types. SD, standard difference; IV, inverse variance; CI, confidence interval; DVA, distant visual acuity; IOL, intraocular lens. Experimental and control indicate trifocal and bifocal IOLs, respectively. [file Image_6.TIF]

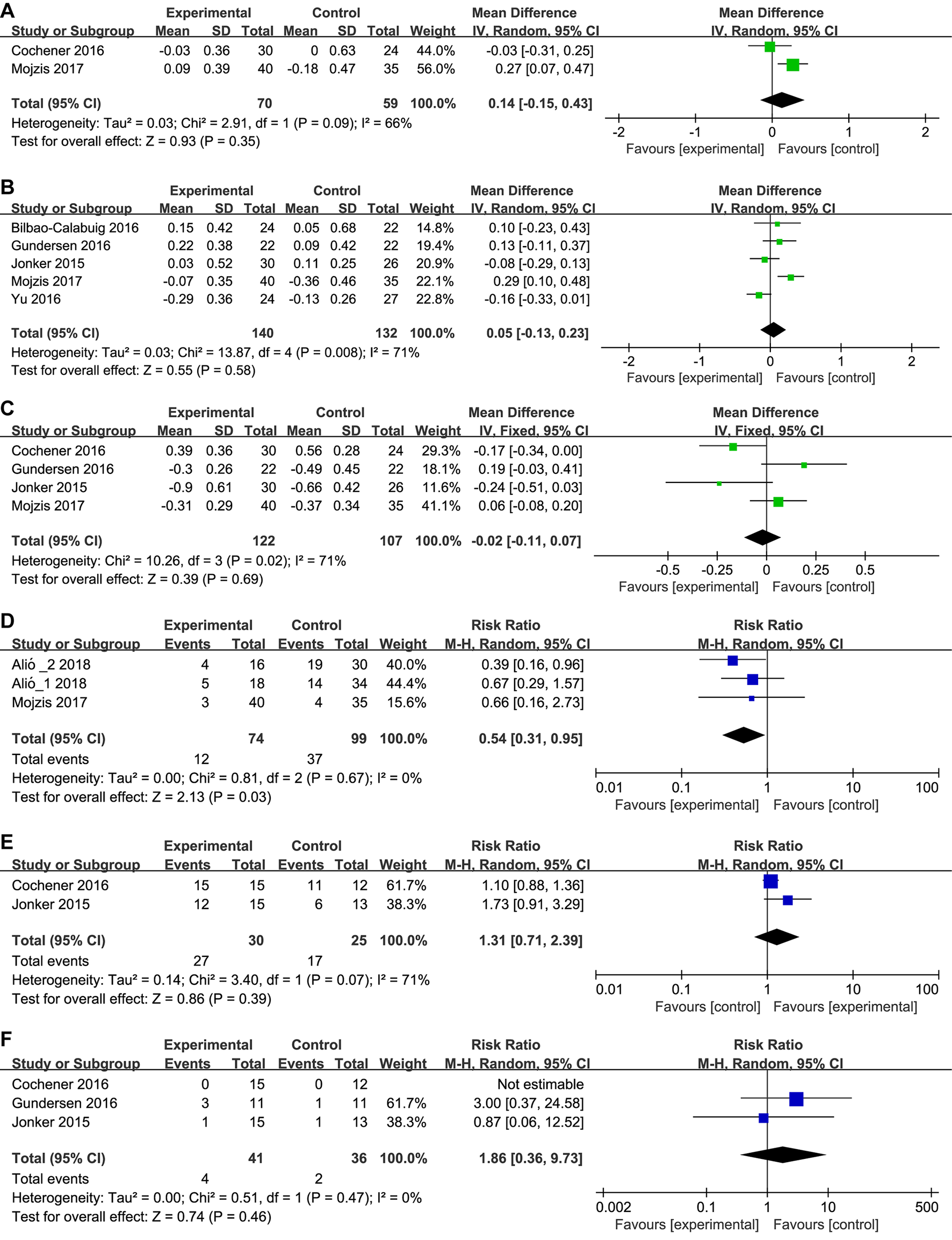

Supplement: Supplementary Figure S7 — Meta-analysis of secondary outcomes including residual sphere (A), spherical equivalence (B), residual cylinder (C), PCO (D), spectacle independence (E), and complications (F). M-H, Mantel-Haenszel; IV, inverse variance; CI, confidence interval; PCO, posterior capsular opacification. Experimental and control indicate trifocal and bifocal IOLs, respectively. [file Image_7.TIF]
